# Simvastatin Inhibits IL-5-Induced Chemotaxis and CCR3 Expression of HL-60-Derived and Human Primary Eosinophils

**DOI:** 10.1371/journal.pone.0157186

**Published:** 2016-06-08

**Authors:** Chia-Hsiang Fu, Wan-Chun Tsai, Ta-Jen Lee, Chi-Che Huang, Po-Hung Chang, Jong-Hwei Su Pang

**Affiliations:** 1 Graduate Institute of Clinical Medical Sciences, College of Medicine, Chang Gung University, Tao-Yuan City, Taiwan, ROC; 2 Department of Otolaryngology, Chang Gung Memorial Hospital, Tao-Yuan City, Taiwan, ROC; 3 Department of Respiratory Therapy, College of Medicine, Chang Gung University, Tao-Yuan City, Taiwan, ROC; 4 Department of Physical Medicine and Rehabilitation, Chang Gung Memorial Hospital, Linkou, Taoyuan City, Taiwan, ROC; Universidade Federal do Rio de Janeiro, BRAZIL

## Abstract

IL-5-induced chemotaxis of eosinophils is an important feature of allergic airway inflammatory diseases. Simvastatin, a lipid lowering agent, has been shown to exhibit anti-inflammatory and anti-allergic effects. Our aim was to investigate the effect of simvastatin on IL-5-induced eosinophil chemotaxis and its regulatory mechanisms. Eosinophils were derived by treating HL-60 clone 15 (HC15) cells with butyric acid (BA) in an alkaline condition or through direct isolation from human peripheral blood. The expressions of CC chemokine receptor 3 (CCR3) and interleukin (IL)-5 receptors (IL5Rα and β) were analyzed using RT/real-time PCR. The granular proteins were stained using fast green. Eotaxin-induced chemotaxis was measured using a transwell migration assay. CCR3 protein expression was revealed by immunocytochemistry. An animal model of allergic rhinitis was established by challenging Sprague–Dawley^®^ rats repeatedly with ovalbumin. Butyric acid significantly increased the expression of IL5Rα and IL5Rβ, CCR3 and granular proteins in HC15 cells, indicating the maturation of eosinophils (BA-E cells). IL-5 further enhanced the CCR3 expression at both the mRNA and protein levels and the eotaxin-induced chemotaxis of BA-E cells. Simvastatin inhibited the effects of IL-5 on BA-E cells, but not in the presence of mevalonate. Similar results were also exhibited in human primary eosinophils. In vivo animal studies further confirmed that oral simvastatin could significantly suppress the infiltration of eosinophils into turbinate tissues of allergic rats. Therefore, simvastatin was demonstrated to inhibit IL-5-induced CCR3 expression and chemotaxis of eosinophils mediated via the mevalonate pathway. We confirmed that simvastatin also reduced eosinophilic infiltration in allergic rhinitis.

## Introduction

Atopic diseases including allergic rhinitis, asthma and atopic dermatitis are global health problems resulting in significant comorbidity, and the economic impact is under-estimated. Allergic rhinitis can increase the recurrence rate of sinusitis and nasal polyps [1], and is a risk factor for asthma development [2]. In IgE-mediated diseases, such as allergic rhinitis and asthma, eosinophils play an important role in the allergic reaction, with their activation and migration into tissues being common features. Activation of eosinophils results in inflammation, tissue edema, airway remodeling, mucus production, and airway hyper-reactivity. Besides, release of several cytokines and chemokines also relates to recruitment of eosinophils, causing corresponding tissue damage [3]. In addition to responding to IL-5 producing cells in allergic reaction, eosinophils can express major histocompatibility complex class II and act as antigen presenting cells in allergic airway [4]. Clinical manifestations of atopic airway diseases and the disease severity are related to accumulation of eosinophils and release of their granular proteins [5]. Interception of their activation, accumulation and degranulation is believed to have a marked therapeutic effect on atopic diseases. Distinct responses to standard therapeutic plan for atopic airway diseases have been reported for eosinophilic and non-eosionophilic airway inflammation, and novel treatments have targeted inflammations based on phenotypes [6].

There are less than 4% eosinophils in human peripheral blood, necessitating large quantities of blood for eosinophils studies to be conducted. HL-60 clone 15 (HC15) cells, derived from a leukaemia cell line, can be induced to differentiate into eosinophils after treatment with butyric acid in mildly alkaline conditions for 5–7 days [7]. Given the eosinophilic phenotype, these cells can respond to eosinophilic chemoattractants and produce eosinophil granular proteins too [8]. Therefore, these cells can be used as an alternative cell model to investigate the behaviors of human eosinophils.

The trafficking of eosinophils into allergic inflammatory sites has been shown to involve several cytokines (e.g. IL-4, IL-5, IL-13) [9], adhesion molecules (e.g. integrins, selectins, intercellular adhesion molecule-1) [10] and chemokines (e.g. RANTES and eotaxins) [11]. Among these cytokines, only IL-5 and eotaxins are selectively specific in regulating eosinophils [12], making them more suitable targets for the study of eosinophil activities. Eotaxin, a potent chemoattractant of eosinophils, binds to CC chemokine receptor 3 (CCR3), which is expressed in cells important in allergic inflammation, and appears potentially crucial for atopic diseases [13]. IL-5, a key cytokine, which binds to the IL5R on eosinophils, is important for the survival, activation and migration of eosinophils [14]. IL-5-induced chemotaxis of eosinophils has been reported to involve several airway diseases [15–18]. Antagonists of IL-5 and CCR3 have been found to have marked potential for inhibition of eosinophil recruitment in allergic diseases [9]. Accordingly, these two receptors are closely associated with eosinophil functions and were investigated in the present study.

Statins, inhibitors of 3-hydroxy-3-methylglutaryl-CoA (HMG-CoA) reductase, are generally utilized as cholesterol-lowering agents. Previous literature has demonstrated their additional anti-inflammatory and immunomodulatory effects [19]. Statin treatment has been shown to reduce asthmatic airway inflammation in *in vivo* murine models [20–21], inhibit monocytes chemotaxis *in vitro* [22] and decrease cell count and cytokine production in human airway secretion [23]. Another recent clinical trial using oral statins to treat asthma, as supplementary therapy to inhaled corticosteroids, showed an additive effect on the inhibition of sputum eosinophils [24]. Through an adequate dose and delivery method, statins may have a potentially therapeutic role in eosinophil-related allergic airway diseases. One of the most commonly used statins, simvastatin, was investigated in the present study using both a HC15 cell model and human peripheral eosinophils. The effect of simvastatin on IL-5-induced CCR3 expression and chemotaxis was examined. An allergic rhinitis animal model was also developed to confirm the *in vivo* effect of simvastatin on eosinophil infiltration. We believe this study may advance the therapeutic principles related to allergic airway diseases.

## Materials and Methods

### Reagents

Butyric acid (BA), simvastatin, mevalonate, ovalbumin (OVA), fast green solution, neutral red, Sirius Red, RPMI-1640 medium and propidium iodide (PI) were purchased from Sigma-Aldrich (St. Louis, MO). Anticoagulant citrate dextrose solution, formula A (ACD-A) was provided by Harvest Technologies Corp (Plymouth, MA). Recombinant human IL-5 was obtained from R&D systems (Minneapolis, MN). Ficoll-Paque^™^, RBC lysis buffer and Liu stain were purchased from Blossom biotechnologies, Toolsbiotech Inc. and Giantech (Taiwan), respectively. Eotaxin and aluminium hydroxide gel were obtained from InvivoGen (San Diego, CA) and TRIzol reagent from Invitrogen (Carlsbad, CA). M-MLV reverse transcriptase was obtained from USB Corporation (Cleveland, OH). Antibodies used for ERK and p38 MAPK in Western blotting were purchased from Cell Signaling Technology (Danvers, MA). Antibodies used in immunocytochemical staining, CCR3 primary antibody and fluorescein isothiocyanate (FITC)-conjugated secondary antibody were obtained from Aviva systems biology (San Diego, CA) and Jackson ImmunoResearch Inc. (West grove, PA), respectively.

### Cell cultures

HC15 cells were obtained from Bioresource Collection and Research Center (Taiwan, ROC). Cells were cultured in RPMI-1640 medium containing 10% fetal bovine serum (FBS) and pH was adjusted to 7.6–7.8 to maintain the differentiation ability towards eosinophils. Cells at 1×10^6^ cells/ml were sub-cultured in a 1:5 dilution in fresh growth medium. Medium was refreshed every 2 to 3 days. Cells were maintained at 37°C in an atmosphere of 95% air/5% CO_2_. The eosinophilic differentiation was induced by treating HC15 cells with 0.5 μM butyric acid for 5 days (BA-E cells). Cell viability was determined by tryptan blue exclusion assay. Cells were mixed with 1/10 volume of 0.4% trypan blue in phosphate-buffered saline, pH 7.2, loaded on a hemacytometer and examined under a microscope at low magnification. If cells took up trypan blue and cytosol appeared in blue, they were considered non-viable. Cell survival rate was calculated as the number of viable cells divided by the total number of cells.

Human primary eosinophils were isolated from the peripheral blood of healthy donors (2 females and 3 males, without any reported allergic disease or taking any medications) with informed consent approved by the Institutional Review Board of Chang Gung Memorial Hospital (104-4615B). In brief, venous blood anticoagulated with ACD-A was processed for centrifugation in combination with Ficoll-Paque^™^. The lower layer was mixed with RBC lysis buffer to lyse the red blood cells and the remaining granulocytes were processed further using a human Eosinophil Enrichment Kit (Stem Cell Technologies, Vancouver, BC). The characteristics of eosinophils such as deep-purple-colored cytoplasmic granules and a bilobed nucleus following staining with Liu stain were verified by morphological observation under a light microscope. Human primary eosinophils were placed in the RPMI-1640 medium containing 10% FBS and immediately used for the experiments.

### Staining of cellular granular proteins

Fast green and neutral red were used to stain the morphological changes and the cellular granular proteins. Cells were cytospun to glass slides, air-dried and fixed in methanol. Slides were incubated in 0.2% fast green solution for 10 min. After washing with running water, slides were stained with 0.5% neutral red for 5 min, then washed again, air-dried and mounted. Cells were observed and photographed using a light microscope. Green color represented cytoplasmic granule proteins and red color represented the nuclei of eosinophils.

### Chemotaxis assay

Eosinophil chemotaxis assays were performed using transwell filters (8μm pore size; Corning, NY) with 10 ng/ml eotaxin used as a chemoattractant along with 650 μl RPMI 1640 added in the lower chamber. After experimental treatments, 2×10^5^ cells were harvested, washed twice with 1× PBS and then resuspended in 250 μl RPMI 1640 in the upper chamber. Chemotaxis assays were performed at 37°C in an atmosphere of 95% air/5% CO_2_ for 3 h. The filter was removed, stained and cell number was counted in four random fields (200×) under microscopy.

### RNA extraction and RT/real-time PCR

Cells were lysed in 0.5 ml TRIzol reagent, and 100 μl chloroform–isoamyl alcohol (49:1, v:v) was added to the homogenate. After vortexing for 1 min, the solution was centrifuged at 12,000 rpm for 20 min at 4°C. The RNA was precipitated by the addition of 0.5 ml isopropanol and kept at −80°C for 1 h. RNA was pelleted by centrifuging the solution at 12,000 rpm for 20 min at 4°C. The RNA pellet was rinsed in ice-cold 75% ethanol, air-dried and dissolved in DEPC-treated ddH_2_O. The cDNA was synthesized from total RNA using M-MLV reverse transcriptase. Real-time PCR was performed with universal cycling conditions (15 min at 95°C, followed by 40 cycles of 30 s at 95°C, 1 min at 55°C and 30 s at 72°C) using an Mx3000 real-time PCR detection system (Agilent Tech, CA, USA) with IQTM SYBR Green Supermix (Bio-Rad Labs, LA, USA) according to the manufacturer’s instructions. GAPDH was used as an internal standard. Oligonucleotide sequences for primers in this study were as follows: GADPH (forward: 5′-GACCTGACCTGCCGTCTA-3′; reverse: 5′-AGGAGTGGGTGTCGCTGT-3′); CCR3 (forward: 5′- TCCCTCTGCTCGTTATGG-3′; reverse: 5′-GATGCTTGCTCCGCTCAC-3′); IL-5 receptor (IL5R): (forward: 5′-ATTGAAGGAACTCGTCTC-3′; reverse: 5′-CTCTCACTTGAACATCGTA-3′).

### Western blotting

HC15 cell extracts were prepared in lysis buffer containing Tris-HCl (pH 7.5), 150 mM NaCl, 1 mM EDTA, 2 mM DTT, 2 mM PMSF, and 1% Triton X-100 (Sigma-Aldrich, St. Louis, MO). Protein concentration of the cell extracts was determined by Bradford assay (Bio-Rad Laboratories, CA). Samples with identical protein quantities were then separated by 10% sodium dodecyl sulfate polyacrylamide gel electrophoresis (SDS-PAGE) and transferred onto a PVDF membrane. The membrane was incubated at room temperature in blocking solution (1% BSA, 1% goat serum in PBS) for 1h, followed by 2-h incubation in blocking solution containing an appropriate dilution of primary antibodies. After washing, the membrane was incubated in PBS containing secondary antibodies conjugated with horseradish peroxidase (Sigma-Aldrich, St. Louis, MO) for 1h. The membranes were washed, and the positive signals developed with enhanced chemiluminescence reagent (Amershan Pharmacia Biotech, Little Chalfont Buckinghamshire, UK). The semiquantitative measurement of the band density was calculated by Digital Analysis Software (Kodak Digital Science TM, Eastman Kodak, Rochester, NY). The band density of each protein was normalized to relative band density of tubulin.

### Immunocytochemical stain

Cells were cytospun on glass slides, air-dried and fixed in 10% formaldehyde for 15 min. Slides were incubated quickly in blocking buffer (Bio-cando, Taipei, Taiwan) for 1 min and then CCR3 primary antibody (1:500 dilution; rabbit) was added for 2 h at room temperature. After washing slides with 1× PBS containing tween-20 (PBST), FITC-conjugated (1:200 dilution) goat anti-rabbit secondary antibody was added for 30 min at room temperature followed by another wash and PI (1:1000) staining for 5 min at 37°C. Finally the slides were washed with PBST, air-dried and mounted with fluorescent mounting medium. Cells were observed using a fluorescent microscope (Nikon DXM1200), and Nikon ACT-1 image software was used for data analysis.

### Allergic rat model

Animal studies were conducted with the approval of the Institutional Animal Care and Use Committee of Chang Gung University (CGU13-074). This study was carried out in adherence to the National Institutes of Health guidelines. We anesthetized the rats by 4–5% inhaled isoflurane for induction and kept 3% isoflurane for maintenance to reduce the distress and suffering of animals before any procedure that is potentially stressful. Humane endpoints and euthanized animals prior to the endpoint of these experiments were applied. We determined when the animals should be euthanized by the signs of anorexia, weight loss more than 20%, dysphagia, dyspnea, cyanosis, or seizures. Overdose sodium pentobarbital would be applied as the method of euthanasia. The health of the rats was examined and monitored every 2 h, and there were no unexpected deaths among the experimental rats.

Male pathogen-free Sprague–Dawley^®^ (SD) rats (BioLASCO Taiwan Co., Ltd., Taiwan), weighing 150 to 250 g, were housed in a temperature- and light-controlled room with free access to food and water. Rats were sensitized and challenged with OVA according to a previous study with some modifications [25]. Rats were sensitized by subcutaneous injection of 1 ml saline containing 1 mg OVA (2 * 10 μg/0.1 ml) and 3.5 mg aluminium hydroxide gel (2%) on day 1. Fourteen days after OVA sensitization, rats from all groups were prepared for allergen challenge. The rats were divided into a control group, sensitized group and treatment group. The sensitized and treatment groups were sensitized by OVA on the first day, whereas the control group was injected with PBS only. Rats from the treatment group were treated with 40 mg/kg simvastatin intragastrically one day before the allergen challenge. Rats were exposed to an aerosolized 0.5% (wt/vol) OVA challenge for 30 min daily on 3 consecutive days, using a nebulizer (PARI BOY^®^, Germany) in a 40 × 50 × 60 cm exposure chamber, with an airflow rate of 4.41 L/min and mean air particle diameter of 3.7 μm. Rats were sacrificed on the next day after the 3-day challenge was completed.

### Histological examination

Turbinate tissue, documented to be the site for a higher infiltration of eosinophils [25], was harvested from the lateral nasal wall, washed with PBS twice, fixed in 10% neutral-buffered formalin for 24 h at room temperature and embedded in paraffin. Sections were cut at 5 μm thickness at the head of turbinates and stained with hematoxylin and eosin (H&E) for routine morphology and eosinophil counts. Sirius Red stain was applied as well for its better eosinophilic staining [26]. The number of infiltrated eosinophils for each group was recorded in five random areas by light microscopy.

### Statistical analysis

A Mann–Whitney test was used for comparison of CCR3 presentation and migrated/infiltrated cell counts between each group. Data were presented as mean ± standard error of mean (SEM). All the *p*-values were 2-tailed, whereas *P* < 0.05 was considered statistically significant and *P* < 0.01 indicated a more marked significance. Statistical analysis was performed using Prism 5.0 (GraphPad Software Inc., La Jolla, CA).

## Results

### Up-regulated expression of IL5R, CCR3 and granular proteins in BA-E cells

**Fig 1:**
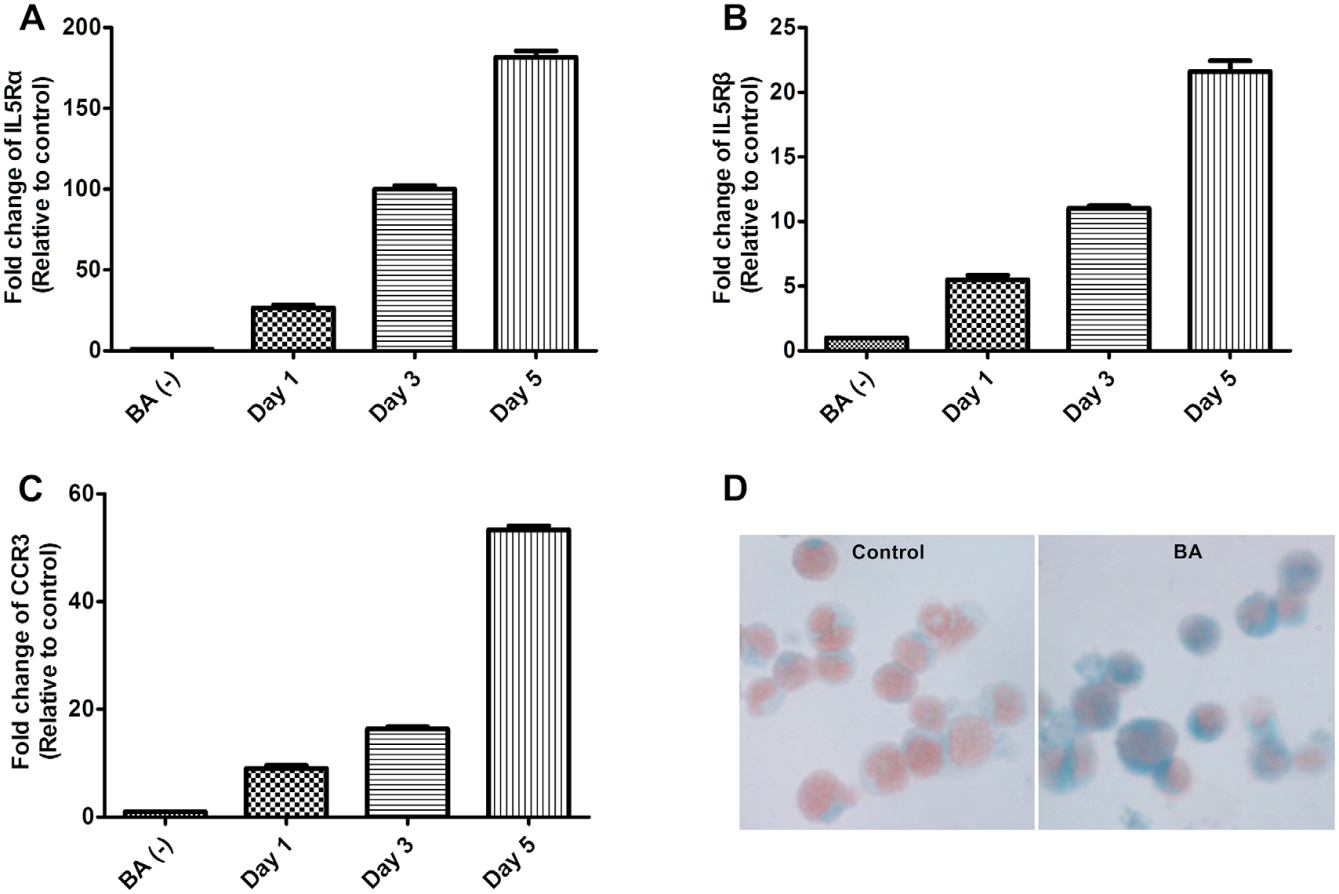
Effects of butyric acid on HC15 cells. RNA was isolated from HC15 cells at different days after 0.5 μM BA treatment and analysed by RT/real-time PCR to detect the expression of (A) IL5Rα, (B) IL5β and (C) CCR3. Data were presented as the combined mean ± standard error of mean (SEM) of n = 4 independent experiments. (D) Fast green and neutral red stain revealed the formation of granular proteins in HC15 cells without (left panel) and with the BA treatment for 5 days (right panel).

HC15 cells were treated with 0.5 μM BA for 0, 1, 3 and 5 days and the expression of IL5Rα, IL5Rβ and CCR3 was analyzed by RT/real-time PCR. The mRNA expression level of IL5Rα was increased significantly by BA and reached its nadir on day 5 (Fig 1A). The mRNA expression levels of IL5Rβ and CCR3 were similar to IL5Rα (Fig 1B and 1C, respectively). Results demonstrated that BA treatment for 5 days successfully induced the expression of corresponding receptors for major eosinophilic chemoattractants including IL-5 and eotaxins. In addition, the cytosolic eosinophilic granular proteins as revealed by fast green stain, were also significantly increased in BA-E cells (Fig 1D). Thus, the differentiation of HC15 cells towards eosinophils by BA treatment for 5 days was suitable for the study of eosinophilic chemotaxis.

### IL-5 enhanced chemotaxis and CCR3 expression in BA-E cells

**Fig 2:**
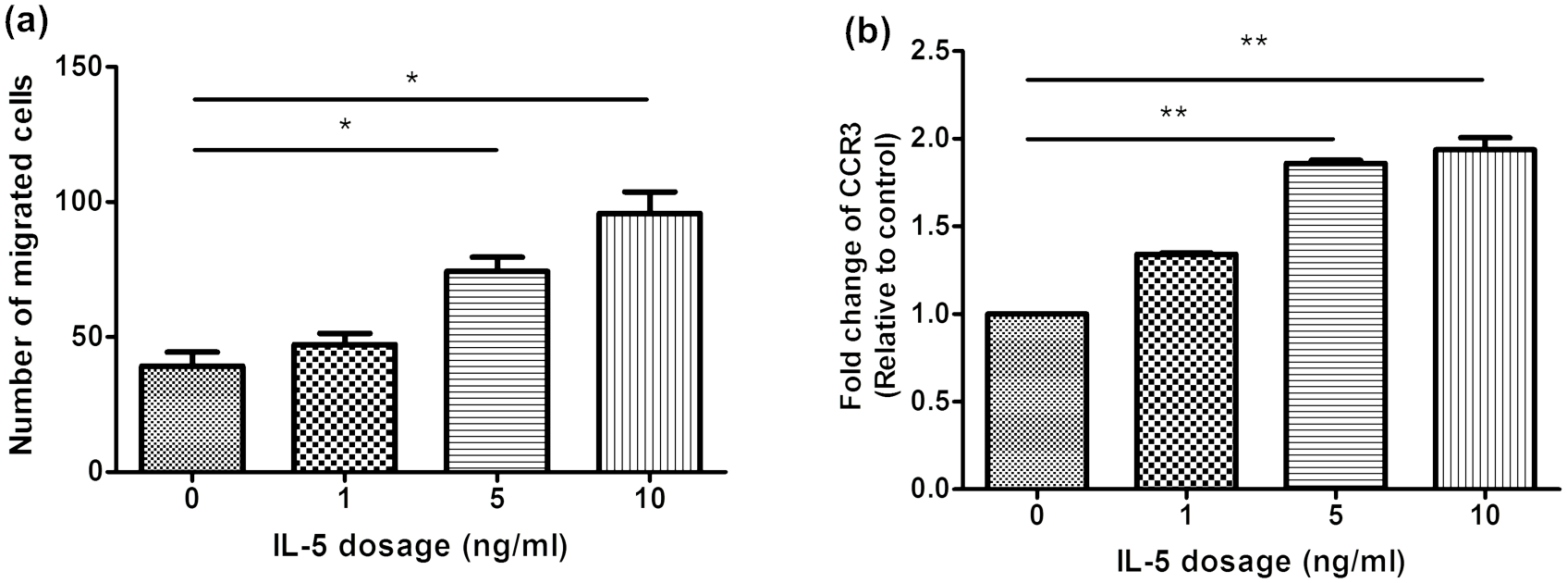
Dose-dependent effect of IL-5 on the chemotaxis and CCR3 expression in BA-E cells. BA-E cells were treated with different concentration of IL-5 for 24 h and (A) the number of migrated cells towards 10 ng/ml eotaxin was determined and (B) the CCR3 expression was analysed by RT/real-time PCR. Data were presented as the combined mean ± SEM of n = 4 independent experiments. *P < 0.05, **P < 0.01, Mann–Whitney U test.

BA-induced expression of both IL5Rα and IL5Rβ enabled the cells to respond to exogenous IL-5, particularly the eosinophilic chemotactic ability. We treated BA-E cells with different concentrations of IL-5 (0, 1, 5, 10, 20 ng/ml) for 24 h and assessed the chemotaxis of cells towards eotaxin stimulation. Fig 2A revealed a dose-dependent enhancement of chemotatic ability and the maximal effect of IL-5 was observed at a concentration of 10 ng/ml. The expression of CCR3, the receptor for eotaxins, was also increased by IL-5 (Fig 2B) with maximal effect at 10 ng/ml similar to the result of the chemotaxis study.

### Simvastatin Inhibited IL-5-Induced Chemotaxis of BA-E Cells

**Fig 3:**
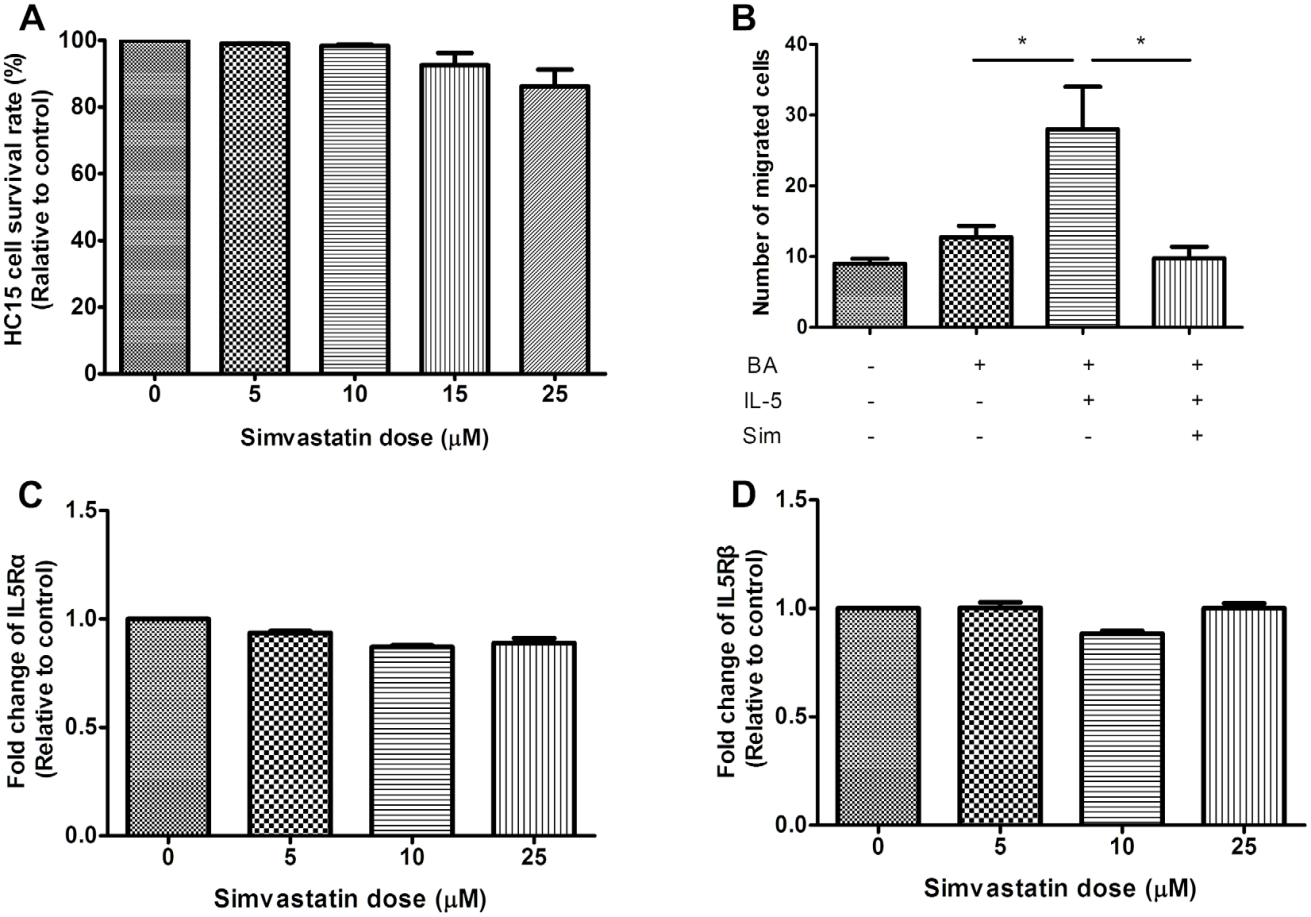
The inhibitory effects of simvastatin on cell viability, chemotaxis and receptor expressions of BA-E cells. BA-E cells were pretreated with simvastatin for 3 h and then stimulated by 10 ng/ml IL-5 for 24 h. (A) The cell survival rate was determined by trypan blue exclusion assay. (B) Chemotaxis assay using 10 ng/ml eotaxin as a chemokine was performed as described to determine the number of migrated cells. Expressions of (C) IL5Rα and (D) IL5Rβ were measured by RT/real-time PCR. Data were expressed as the combined mean ± SEM of n = 4 (A, B) or n = 5 (C, D) independent experiments.*P < 0.05, Mann–Whitney U test.

The effect of simvastatin on IL-5-induced chemotaxis of BA-E cells was investigated. We found a dose-dependant inhibition ability for cell viability for BA-E cells and being favorable if pretreated simvastatin was no more than 25 μM (Fig 3A). Cell viability would decrease to 70% or less if HC15 cells were pretreated with simvastatin more than 25 μM. HC-15 derived eosinophils were pretreated with 25 μM simvastatin for 3 h before stimulation with IL-5 and chemotaxis was measured at 24 h after the addition of 10 ng/ml IL-5. Fig 3B revealed that simvastatin had a significant inhibitory effect on the IL-5-induced chemotactic ability towards eotaxin (*P* = 0.028).

### Simvastatin Effect on IL5R and CCR3

**Fig 4:**
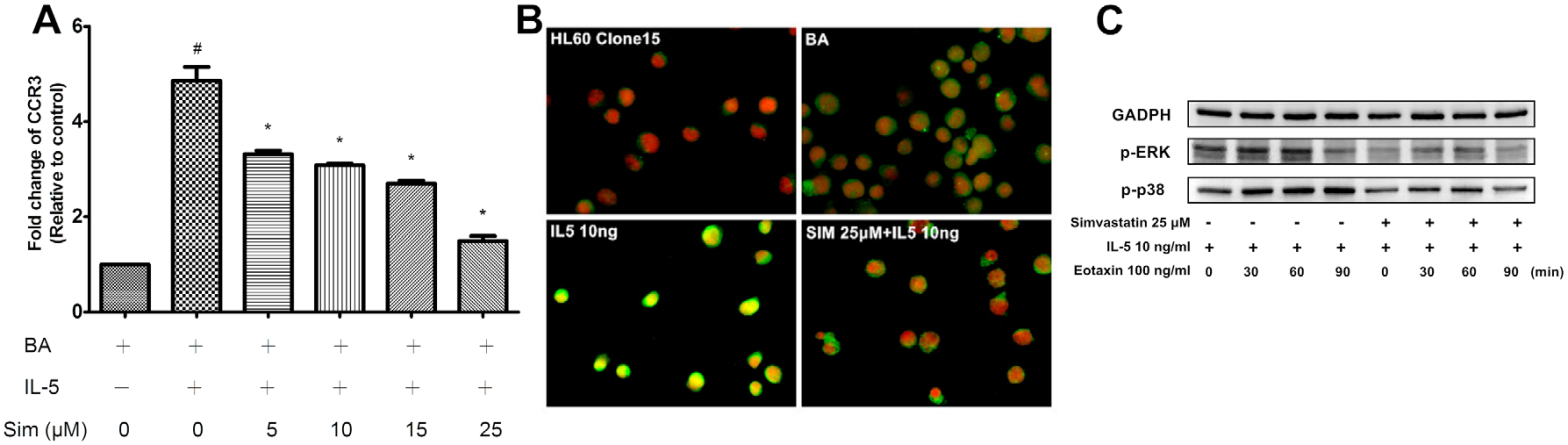
Effect of simvastatin treatment on CCR3 and downstream activation of ERK1/2 and p38 in BA-E cells. (A) BA-E cells were pretreated with 0–25 μM simvastatin for 3 h and stimulated by 10 ng/ml IL-5 for 24 h. Presentation of CCR3 measured by real-time PCR was inhibited by simvastatin treatment in dose-dependant manner if BA-E cells treated with simvastatin no more than 25 μM. (B) CCR3 expression (green) on the surface of BA-E cells was revealed by immunocytochemical stain in different treatments. (C) BA-E cells were pretreated with/without 25 μM simvastatin for 3 h, followed by 10 ng/ml IL-5 for 24 h and 100 ng/ml eotaxin was added for 0, 30, 60 and 90 min. The activations of ERK1/2 and p38 were analyzed by Western blot with the use of antibodies against phosphorylated ERK1/2 and phosphorylated p38 MAPK. Data were expressed as the combined mean ± SEM of n = 5 independent experiments. #*P* < 0.01, compared with BA-E cells without IL-5 treatment; *P < 0.05, compared with IL-5 primed BA-E cells without simvastatin treatment, Mann–Whitney U test.

The inhibition of IL-5-induced chemotaxis towards eotaxin could possibly be because of the modulation of either IL-5R or CCR3. To understand the mechanism of the inhibitory effect of simvastatin on chemotaxis, the expression levels of IL5R and CCR3 were investigated. Results demonstrated neither IL5Rα nor IL5Rβ expression in BA-E cells was affected by simvastatin treatment for 3 h (Fig 3C and 3D). However, the expression of CCR3 at the mRNA level was down-regulated in a dose-dependant manner as revealed by RT/real-time PCR (*P* = 0.028) (Fig 4A). Similar results were also obtained by immunofluorescent staining of the BA-E cells, showing the inhibitory effect of simvastatin on CCR3 protein expression (Fig 4B).

### Simvastatin inhibited p38 MAPK and ERK1/2 phosphorylation in BA-E cells

To further discover the effect of simvastatin for signaling pathway related to CCR3-mediated chemotaxis of eosinophils, downstream effectors of CCR3 pathway, p38 MAPK and ERK1/2, were investigated. P38 MAPK and ERK1/2 have been proved to involve in the chemotaxis of eosinophils [27]. BA-E cells were co-cultured with / without 25 μM simvastatin for 3 h before 10 ng/ml IL-5 and 100 ng/ml eotaxin stimulation. Western blot was applied to compare the simvastatin effect for the phosphorylation activity of p38 MAPK and ERK1/2. Eotaxin treatment enhanced the presentation of phospho-p38 MAPK (p-p38) and phospho-ERK1/2 (p-ERK) after 30 min (Fig 4C). A significant suppression was discovered for the presentation of p-p38 and p-ERK1/2 in simvastatin-treated BA-E cells. Accordingly, we may propose the suppression of phosphorylation activity of p38 MAPK and ERK1/2 were related to inhibition effects of simvastatin for IL-5 enhanced CCR3-mediated chemotaxis of eosinophils.

### Mevalonate reversed the inhibitory effects of simvastatin on chemotaxis and CCR3 presentation

**Fig 5:**
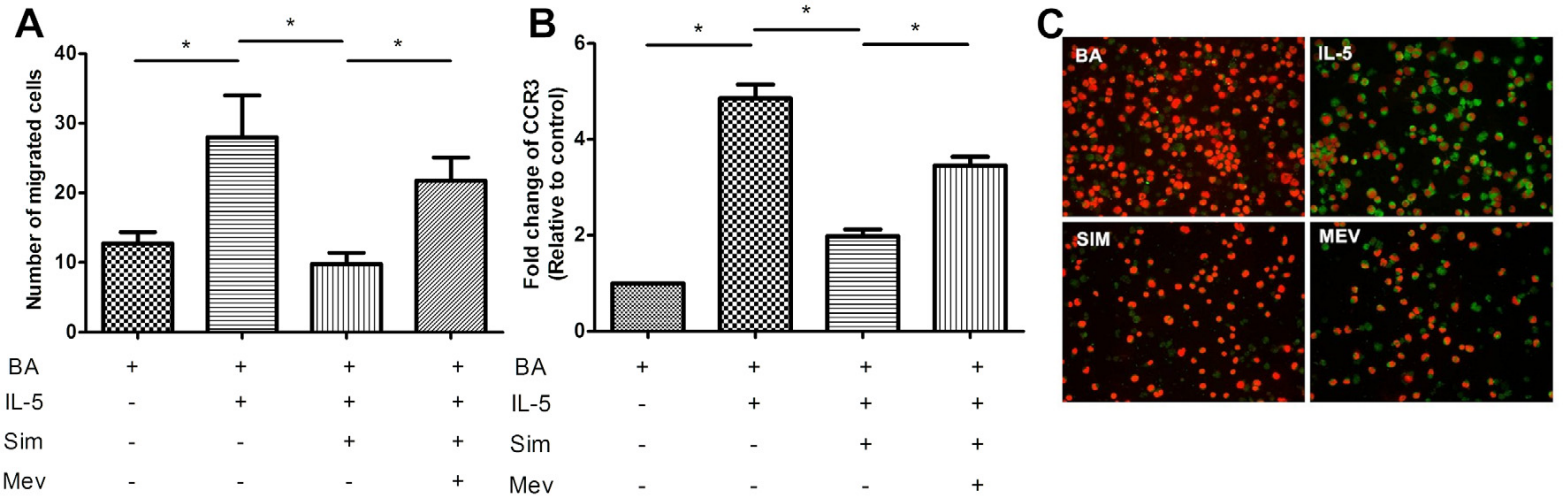
Mevalonate replacement reversed the inhibitory effects of simvastatin on BA-E cells. BA-E cells were pretreated with 2 μM mevalonate and 25 μM simvastatin for 3 h before the stimulation of 10 ng/ml IL-5 for 24 h. (A) Chemotaxis assay using eotaxin as a chemokine was performed as described. (B) CCR3 mRNA expression was analyzed by RT/real-time PCR. Data were expressed as the combined mean ± SEM of n = 4 independent experiments. *P < 0.05, Mann–Whitney U test. (C) CCR3 protein expression (green color) on the surface of BA-E cells was revealed by immunocytochemical stain in different treatments.

Statins can suppress the formation of mevalonate by inhibiting HMA-CoA reductase. To determine whether the inhibitory effect of simvastatin on IL-5-induced chemotaxis was mediated by blockage of the mevalonate pathway, the restorative effect of mevalonate was analyzed. BA-E cells were co-cultured with 2 μM mevalonate and 25 μM simvastatin for 3 h before IL-5 stimulation. A chemotaxis assay and RT/real-time PCR for CCR3 expression were subsequently performed as mentioned above. Results demonstrated that mevalonate could reverse the inhibition of chemotaxis (Fig 5A) and CCR3 expression at both the mRNA and protein levels (Fig 5B and 5C, respectively). Thus, the inhibitory effects of simvastatin for IL-5-induced chemotaxis and CCR3 presentation were achieved by the inhibition of the mevalonate pathway.

### Simvastatin exerted similar effects on human primary eosinophils

**Fig 6:**
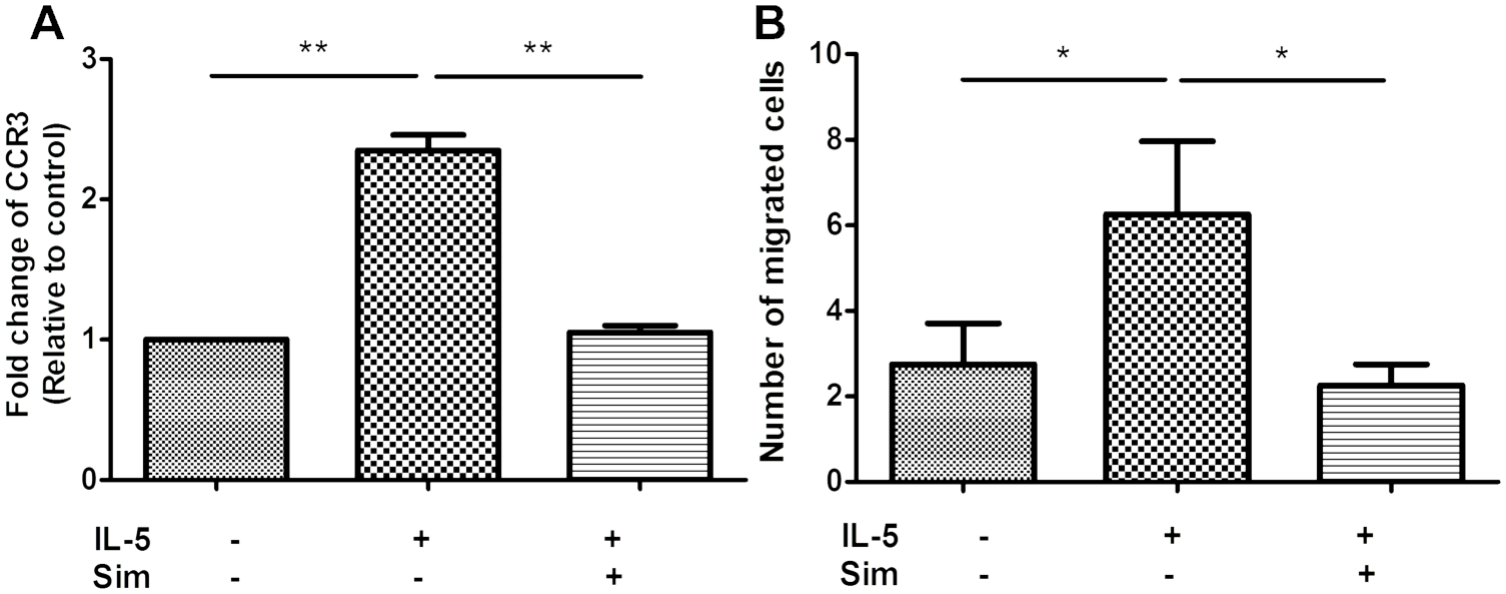
Effects of simvastatin on the chemotaxis and CCR3 expression in human primary eosinophils. Human primary eosinophils isolated from peripheral blood were pretreated with simvastatin 25 μM for 3 h and then stimulated by 10 ng/ml IL-5 for 24 h. (A) CCR3 expression and (B) chemotatic ability towards eotaxin were analyzed. Data were expressed as the combined mean ± SEM of n = 5 (A) or n = 4 (B) independent experiments.*P < 0.05, **P < 0.01, Mann–Whitney U test.

To test whether the modulation of chemotaxis and CCR3 expression by IL-5 and simvastatin in BA-E cells could represent the cellular physiology of primary eosinophils isolated directly from human blood, we performed the same experiments as described above. Human primary eosinophils pretreated with 25 μM simvastatin for 3 h were stimulated by 10 ng/ml IL-5 for 24h, and then the chemotactic ability and CCR3 expression were analyzed with at least 90% cell viability. The expression of CCR3 significantly increased after IL-5 stimulation and was significantly suppressed by simvastatin (both *P* < 0.001) (Fig 6A). Simvastatin also significantly inhibited the IL-5-induced chemotaxis of human primary eosinophils (*P* = 0.012) (Fig 6B), similar to the results observed in BA-E cells.

### Simvastatin reduced eosinophil infiltration in a rat allergic rhinitis model

**Fig 7:**
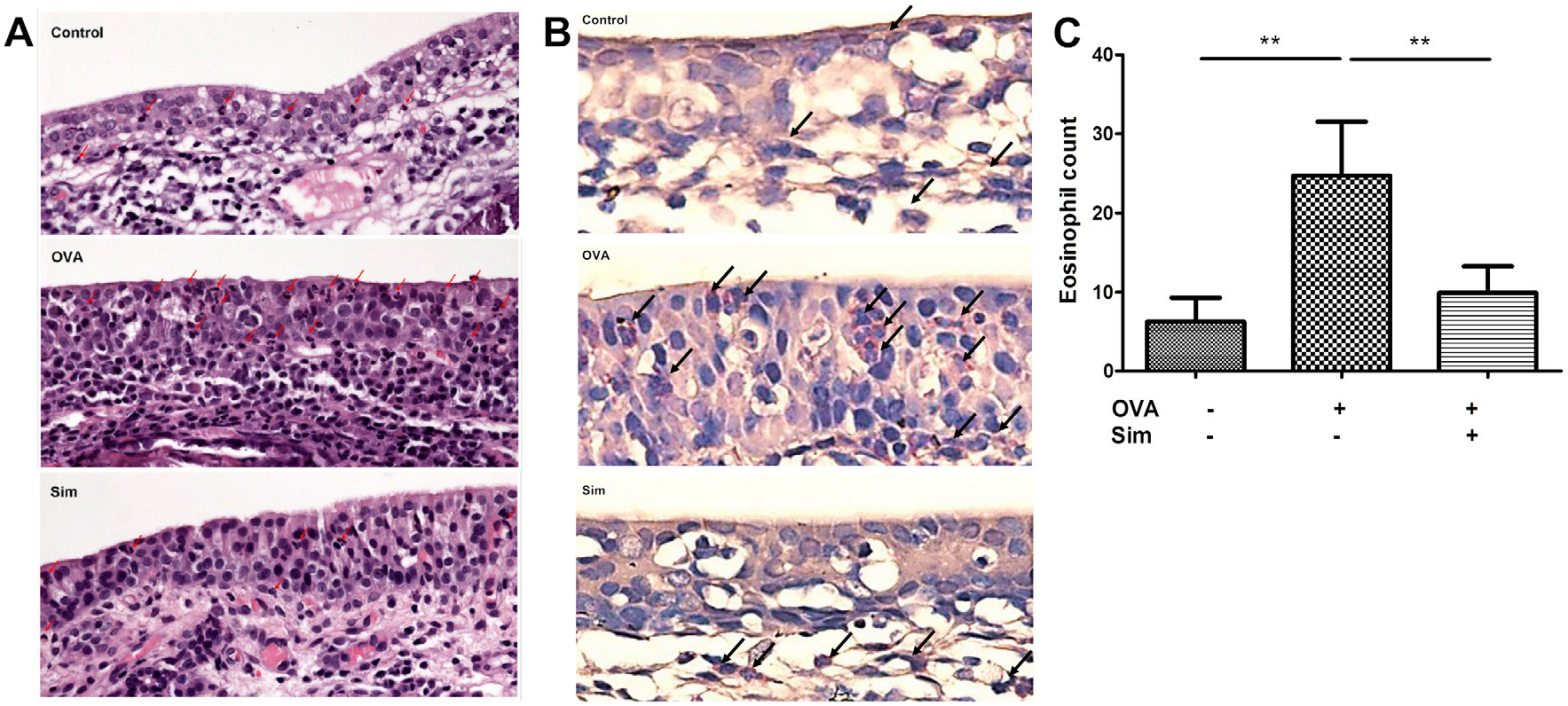
Simvastatin reduced eosinophil infiltration in a rat model of allergic rhinitis. Rats were sensitized by subcutaneous OVA injection on day 1 except for control group. Rats of the simvastatin treatment group were treated with 40 mg/kg simvastatin intragastrically one day before allergen challenge. Fourteen days after OVA sensitization, rats of all groups received allergen challenge with aerosolized OVA for 30 min daily on three consecutive days and sacrificed on the next day to examine the infiltration of eosinophils in turbinate mucosa by (A) H&E stain and (B) Sirius Red stain under light microscopy (400×). (C) Number of eosinophil infiltration counts was recorded in five random areas for each group. Data were expressed as the combined mean ± SEM of five rats. **P < 0.01, Mann–Whitney U test.

The inhibition of IL-5-induced chemotaxis and CCR3 expression in eosinophils by simvastatin may impede *in vivo* tissue infiltration of eosinophils. To explore the *in vivo* effect of simvastatin on eosinophil infiltration, an allergic rhinitis rat model was established. The eosinophil infiltration was observed in turbinate tissue by H&E stain under light microscopy (Fig 7A). The results from 15 rats revealed an increase in the number of infiltrated eosinophils after OVA sensitization and subsequent challenge (*P* = 0.008) (Fig 7C). After simvastatin treatment, the number of infiltrated eosinophils was significantly inhibited (*P* = 0.008). Another eosinophil-specific stain, Sirius Red [26], was applied and revealed the similar results (Fig 7B). Simvastatin was demonstrated to exert an *in vivo* inhibitory effect on reducing the eosinophil infiltration in an animal model of allergic rhinitis.

## Discussion

HL-60 clone 15 cells maintained in an alkaline condition have been demonstrated to differentiate into eosinophils by treatment with butyric acid, a histone deacetylase [28]. The differentiated cell line has been utilized to exhibit ERK1/2 phosphorylation after IL-5 or eotaxin stimulation and human eosinophils had the same response [29]. RNA silencing of GATA-2 had a similar effect, resulting in a decreased expression of eosinophil-derived neurotoxin for both HC15-derived and differentiating human eosinophils derived from CD34+ hematopoietic progenitors [30]. As revealed by various chemical stain methods, HL-60-derived eosinophils can produce typical granules as seen in human primary eosinophils [31]; therefore, they are suitable cell models for human primary eosinophils. It could help us to understand more about the characteristics of eosinophils, which although constitute a small percentage of the human peripheral blood, play a critical role in allergic diseases and potentially contribute to the development of new therapeutic approaches.

A previous literature reported fluvastatin and lovastatin significantly inhibited GM-CSF-stimulated eosinophil adhesion to rhICAM-1 but had no effect for unstimulated eosinophils [32]. In that study, fluvastatin and lovastatin at a low concentration of 1–10 nM significantly inhibited GM-CSF-stimulated eosinophil adhesion to rhICAM-1, but not by same concentration of simvastatin or pravastatin. It clearly indicates that although statins all exhibit the same effect to inhibit HMG-CoA reductase and reduce LDL and triglycerides in the blood, their actions in different cell types and appropriate dosage should be carefully determined by experiments. Further, in inflammatory and allergic disorders, such as asthma and allergic rhinitis, are associated with the behaviors of primed eosinophils. Thus we preceded our study with primed eosinophils stimulated with IL-5, including the following investigation of inhibitory ability for statins.

Because the recruitment of eosinophils is closely associated with the severity of allergic diseases, the chemotaxis of eosinophils and related regulatory mechanisms have become the emphasis of the present study. Among the cytokine receptors that have been identified on eosinophils those specific to eosinophil trafficking include IL5R for IL-5 and CCR3 for eotaxin [16], and these were proved to be highly expressed on HC15-derived eosinophils in this study. Although IL-5-induced chemotaxis has been generally recognized as one of the the most critical roles of eosinophils in severity of atopic airway diseases, the anti-IL-5 therapy targeting elimination of eosinophils has not brought about any major clinical improvements [33]. The present investigation discovered that a maximal effect could be reached for both CCR3 expression and IL-5-induced chemotaxis by treatment with IL-5. Given CCR3 is also one of the chemokine receptors selectively responsible for eosinophil trafficking, its amplification during IL-5 stimulation is likely to be involved in the reinforcement of chemotactic ability for both BA-E cells and human primary eosinophils.

In addition to the cholesterol-lowering effect, statins have been demonstrated to exhibit anti-inflammatory and immunomodulation effects [34–35]. Simvastatin has also been found to significantly reduce the rhinovirus-induced CXCL10 secretion from human alveolar macrophages which corresponded with decreases in IFN-α secretion and pSTAT1 [36]. Rhinovirus infection frequently triggers the exacerbations of asthma and currently no appropriate intervention is available. Simvastatin might be further developed based on this anti-inflammatory effect to attenuate the chance of asthma triggered by rhinovirus. As a potential treatment for respiratory inflammatory diseases, simvastatin is likely to exhibit multiple beneficial effects that required more studies to understand in details. In addition, a decrease in inflammatory infiltrates in lung tissue and cell counts in bronchoalveolar lavage fluid (BALF) has been previously demonstrated in allergic murine models after statin treatment [20–21]. Another mouse model of asthma revealed that a CCR3 monoclonal antibody could significantly suppress airway eosinophilia and mucus production without decreasing IL-5 levels in BALF [37]. Simvastatin in the present study, for the first time, we demonstrated that simvastatin could significantly inhibit IL-5-induced chemotaxis of BA-E cells with comparable cell viability. Compared to asthma, allergic rhinitis is a more prevalent disease with quite different pathogenesis. We also firstly proved that simvastatin can inhibit the eosinophil infiltration in nasal turbinates of an allergic rhinitis animal model that is different from previous studies focused only in lung or BALF of asthmatic mice [20, 21]. The expression of CCR3, but not IL5R, was suppressed by simvastatin as well both at the mRNA and protein levels in BA-E cells or human primary eosinophils. The inhibition of CCR3, one of the most specific chemokine receptors responsible for eosinophil trafficking, may contribute significantly to the suppression of inflammatory cell recruitment in eosinophil-dominant allergic diseases. In addition, our in vitro study suggests that the molecular mechanism underlying this inhibitory effect of simvastatin is likely associated with the suppression of downstream activation of ERK1/2 and p38 signaling pathways in IL-5-treated eosinophils. This pharmacological effect of simvastatin has not yet been reported before either. We may propose the phosphorylation activity suppression was related to inhibition effects of simvastatin for IL-5 enhanced CCR3-mediated chemotaxis of eosinophils. Simvastatin has been examined in only one previous study on the expression of CCR3 in murine tracheal epithelium cells [37]. However, this study only investigated the genes that could be induced by IL-13. Because the expression of CCR3 in murine tracheal epithelium cells was not affected by IL-13, the effect of simvastatin was not observed. Therefore, the modulation of CCR3 expression is dependent on the cell type and cytokine used in the study. Our results demonstrated the specific inhibitory effect of simvastatin on CCR3 expression in eosinophils.

In eosinophilic airway diseases, statins have been shown to have no steroid-sparing effect for asthmatics [35]. However, they are useful in attenuating Th2 cytokines concentration in BALF [38] and decreasing the eosinophil counts in sputum related to the severity of asthma [24] and hospitalization for asthma attacks, which was shown in a nationwide study [39]. Furthermore, the inhibitory effect of statins has been proposed regarding the proliferation, myofibroblast differentiation and collagen production in nasal polyp-derived fibroblasts [40–41], indicating that statins may be another potential treatment for nasal polyps, which is a Th2-dominant and eosinophil infiltrative sinonasal disease [42]. We demonstrated IL-5-induced CCR3 gene expression of eosinophils is already reduced by simvastatin at the concentration of 5 μM when cell viability was not yet affected (Fig 4A). The clinical usage of simvastatin for an adult is suggested to be 10–40 mg/day. Dosage at 80 mg/day is sometimes applied. The blood volume in an adult is 60–80 ml/Kg. Therefore, for a 60 Kg adult, the maximum concentration of simvastatin is calculated to be around 10–30 μM. Xu *et al* have reported the effect of simvastatin delivered by inhalation at a higher concentration of 5 mg/ml (12 mM) to attenuate airway inflammation in a murine model of asthma [43]. By delivering simvastatin via inhalation could eliminate the problems of systemic adverse effects and low clinical efficacy by oral administration. These results suggest that simvastatin is a potential anti-inflammatory drug for airway inflammatory diseases with properties suitable for delivery by inhalation, which probably could also be applied to treat allergic rhinitis. Recently, a novel simvastatin inhalation formulation is developed and characterized by Tulbah *et al* [44]. In this delivery method, simvastatin at concentration as high as 0.5%, w/w (10 mM) is formulated. Therefore, study on the local effect of simvastatin at higher concentration is necessary in the future for the development of new inhalation method for airway inflammatory diseases.

Co-treatment with mevalonate could reduce the inhibitory effect of statins for pulmonary inflammation and leukocyte influx into airways in an allergic murine model [45]. In this study, mevalonate replacement reversed the inhibitory effect of simvastatin on IL-5-induced chemotaxis, thus statin-induced inhibitory effects on eosinophil chemotaxis may occur through the mevalonate pathway. It also neutralized the reduction of CCR3 expression by simvastatin in both mRNA and protein expression. Because CCR3 is crucial for IL-5-induced chemotaxis as shown previously in this study, mevalonate reversed the inhibitory effect of simvastatin on chemotaxis probably by way of restoring the expression of CCR3.

Collectively, CCR3 is of great value in modulating IL-5-induced chemotaxis, a key step in the allergic reaction, in both BA-E cells and human primary eosinophils. Simvastatin, a potentially useful therapeutic agent, with its inhibitory effect on chemotaxis of eosinophils via the mevalonate pathway may be quite beneficial in eosinophil-predominant atopic diseases and may provide another treatment option for those unresponsive to anti-IL5R therapy. The molecular mechanism underlying this inhibitory effect of simvastatin is possibly associated with the suppression of CCR3 gene expression and downstream activation of ERK1/2 and p38 signaling pathways in IL-5-treated eosinophils that need more investigations to confirm in the future. The present study also provided important evidence about BA-E cells that behave similar to human primary eosinophils in response to IL-5 including the CCR3 expression and the chemotaxis towards eotaxin, a crucial step in allergic reactions. Therefore, BA-E cells could be used alternatively as an excellent cell model for exploring the molecular regulation of eosinophilic functions and progressing the development of potential therapeutics for atopic diseases.
